# Bifunctional N-TiO_2_/C/PU Foam for Interfacial Water Evaporation and Sewage Purification

**DOI:** 10.3390/ma18071550

**Published:** 2025-03-29

**Authors:** Ke Wang, Weifeng Li, Yumei Long

**Affiliations:** 1 College of Chemistry, Chemical Engineering and Materials Science, Soochow University, Suzhou 215123, China; kewang00203@163.com; 2 The Key Lab of Health Chemistry and Molecular Diagnosis of Suzhou, Soochow University, Suzhou 215123, China

**Keywords:** solar interfacial evaporation, photothermal, photocatalytic, TiO_2_

## Abstract

As an environmentally friendly and clean energy technology, solar-driven interfacial evaporation technology has attracted wide attention. However, organic pollutants can easily pollute distilled water during the evaporation of wastewater. In this work, we report a strategy of N-TiO_2_/C solar absorption with a low bandgap (2.33 eV), excellent light absorption ability, and high photothermal conversion efficiency (48.2%). Black N-TiO_2_/C was prepared by the sol-gel method in the presence of hexamethylenetetramine as a source of nitrogen and carbon. The simultaneous N doping and C with superior photothermal effect rapidly increased the surface temperature of the material, reduced the recombination rate of electrons and holes, and improved the photocatalytic activity, showing great potential for solar thermal energy conversion. The prepared solar absorbent and polyurethane (PU) were mixed evenly to form a porous N-TiO_2_/C/PU (NTCP) foam for purifying water. The evaporator produced clean water at a rate of 1.73 kg m^−2^ h^−1^ under the simulated sunlight of 1 sun irradiation. Meanwhile, the evaporator simultaneously photodegraded methylene blue (MB) and rhodamine B (RhB) underwater at a removal rate > 90%. The bifunctional solar water evaporation device combining photocatalytic and photothermal effects holds great potential for water purification.

## 1. Introduction

With rapid population expansion and industrial development, the serious shortage of clean water has become a bottleneck problem worldwide [1,2]. Up to now, tremendous efforts have been made to explore cost-effective technology for water purification [3], such as electro-dialysis [4], multiple-effect distillation [5], solar evaporation [6], photocatalytic degradation of sewage [7], and adsorption and advanced oxidation processes (AOPs) [8]. Most traditional technologies tend to cause a lot of energy consumption, environmental pollution, and high capital costs for infrastructure construction. Therefore, using a low-cost, sustainable, and renewable technology to produce clean water has become an important development direction. As an ideal strategy, solar-driven interfacial evaporation can utilize solar energy and can selectively heat part of the water, avoiding heating the bulk of the water and wasting photothermal materials [9]. Therefore, this technology has a wide application prospect in achieving sustainable freshwater production. As research progressed, it was also revealed that pollutants in the water may gradually accumulate on the evaporator [10,11,12], which would eventually pollute the photothermal layer and reduce the equipment efficiency. Solar water evaporation with photocatalytic degradation is a promising method of clean water production. However, research is still needed to understand the synergistic effect between solar water evaporation and photocatalysts. It is of great significance to develop a solar evaporation system for simultaneous high-efficiency freshwater production and photocatalytic degradation.

To meet this demand, material design should consider two factors: (1) full-spectrum absorption and excellent photothermal conversion performance and (2) rapid separation of photo-generated carriers. In recent years, many photothermal materials have been fabricated, such as plasma metal [13,14], semiconductor [15,16,17,18], and carbon-based materials [19,20,21]. The semiconductor anatase TiO_2_ has attracted extensive attention in the field of photocatalysis because of its chemical stability, non-toxicity, and superior photocatalytic activity [22,23]. However, the wide band gap (3.2 eV) of TiO_2_ limits its application under solar irradiation [24]. The impurity state of nonmetallic ions is closer to the valence band, so doping nonmetallic ions can better expand the light absorption range [25,26]. For example, Cao’s group prepared a N-TiO_2_ photothermal catalyst and found that N doping provides TiO_2_ with a suitable valence band position (2.51 eV) for the photo-oxidation reaction [27]. Zhang et al. reported that N-doped TiO_2_ (N-TiO_2_) can extend the light absorption range to the visible light region [28] and effectively improve photo-induced charge transfer and separation, resulting in high selectivity photocatalytic oxidation of alcohols. In addition, researchers have confirmed that the combination of TiO_2_ and carbonaceous materials can inhibit electron–hole recombination and increase the absorption capacity of visible and near-infrared (Vis–NIR) light [29]. For example, Wang’s group designed a solar evaporator including TiO_2_ foams and carbon as the photothermal sensitizer [30], which broaden the adsorption region to infrared light. The hybrid photothermal membrane holds great potential for applications in seawater desalination and wastewater treatment. Zha et al. combined N-TiO_2_ with carbon nanotubes (CNTs) using polyvinylidene fluoride (PVDF) as a substrate [31] and reported a solar evaporation rate of 1.35 kg m^−2^h^−1^ and efficient degradation of organic dyes in source water simultaneously. Liu et al. composited photocatalytic black titania with carbon cloth and reported its bifunctional applications with a solar steam conversion efficiency of 94% under 1 sun irradiation and a degradation rate of rhodamine B of about 95% [32].

Here, a black N-TiO_2_/C with high broadband adsorption and high photothermal conversion efficiency (48.2%) was constructed using hexamethylenetetramine (HMT) as a rich carbon and nitride source. The N doping level was effectively modulated by controlling the annealing temperature. Particularly, incorporated with polyurethane (PU), N-TiO_2_/C/PU (NTCP) was prepared, and the evaporator exhibited the advantages of fast water transport, broad absorption spectra, and rapid ability to reach high temperatures on the surface. As a result, it presented an excellent evaporation rate of 1.73 kg m^−2^ h^−1^ and efficiency of 105.8% under 1.0 sun irradiation. Meanwhile, the evaporator effectively degraded organic dyes with a photodegradation rate > 90%. Thus, the combination of bifunctional photothermal and photocatalysis improved the utilization of solar energy and the application of a traditional evaporator.

## 2. Materials and Methods

### 2.1. Materials

Titanic chloride (TiCl_4_, ≥99%) and hexamethylenetetramine (HMT; C_6_H_12_N_4_, ≥99%) were purchased from Sinopharm Chemical Reagent Co., Ltd. (Shanghai, China). Absolute ethanol (≥99%) was provided by Shanghai Ling Feng Chemical Reagent Co., Ltd. (Shanhai, China). Polyurethane raw materials were purchased from Sigma-Aldrich. Rhodamine B (RhB) and methylene blue (MB) were obtained from Aladdin Reagent Company (Shanghai, China). All chemicals used in this work were used as received, without further purification.

### 2.2. Preparation of N-TiO_2_/C

**Figure 1:**
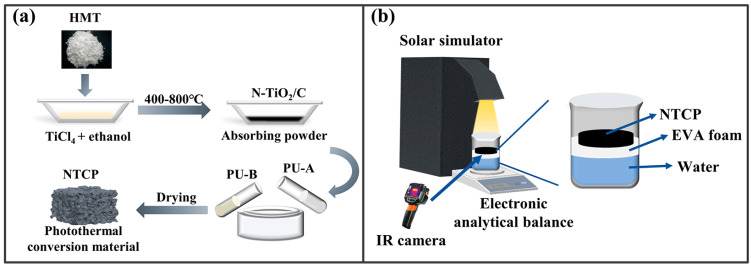
(**a**) N-TiO_2_/C and NTCP synthesis route and (**b**) solar evaporation process.

N-TiO_2_/C composites were obtained by a one-step calcination method using carbon and nitrogen elements in the precursor. The preparation route is shown in Figure 1a, and the specific experimental steps were as follows: Typically, 0.01 mL of TiCl_4_ and a given amount of HMT were first dissolved in 15 mL of absolute ethanol. The mixture was continuously stirred for 3 h and dried in an oven at 60 °C. Finally, the precursor was calcinated at 400 °C in an argon (Ar) atmosphere for 3 h at a heating rate of 10 °C/min to achieve an oxygen-free environment to promote the carbonization reaction. The samples prepared at different temperatures were labeled as N-TiO_2_/C-x (x = 400, 500, 600, and 700).

### 2.3. Preparation of N-TiO_2_/C/PU (NTCP) Foams

The porous polyurethane (PU) foam was prepared by mixing commercially purchased polyurethane A glue and B glue in a mass ratio of 100:40. N-TiO_2_/C powders were mixed in PU foam before forming with vigorous stirring. The NTCP foams with different weights of 0, 10, 30, 50, and 100 mg were prepared, respectively, referring as PU, NTCP-10 mg, NTCP-30 mg, NTCP-50 mg, and NTCP-100 mg. After a few minutes of shape fixation, they were placed in the oven to dry for 10 min at 60 °C.

### 2.4. Characterization

The obtained samples were characterized by X-ray diffraction (XRD, D8 Advance, Bruker, Coventry, UK) using Cu–Kα radiation (λ = 0.15406 nm) from 10° to 80°. The morphology of samples was obtained from scanning electron microscopy (SEM, SU8010, HITACHI, Tokyo, Japan), transmission electron microscopy (TEM, HT7700, HITACHI, Tokyo, Japan), and high-resolution transmission electron microscopy (HRTEM, Talos F200X G2, Thermo Fisher Scientific, Waltham, MA, USA). Elemental mapping was conducted by energy-dispersive X-ray spectroscopy (EDX) during SEM observation. The surface elemental composition was analyzed by X-ray photoelectron spectroscopy (XPS, EXCALAB 250 XI, Thermo Fisher Scientific, Waltham, USA). The Raman spectra were recorded by a high-spectral-resolution confocal Raman Microscope (Raman, LabRAM Soleil, HORIBA, Kyoto, Japan) equipped with a 514 nm laser excitation source. Infrared spectra (FTIR) were acquired with an IR-408 spectrometer (Shimadzu, Kyoto, Japan) in transmission mode. Thermo-gravimetric analysis (TGA) was performed on a TG/DTA thermo-gravimetric analyzer (TGA, NETZSCH STA 449F3, Bavaria, Germany). The absorbance spectra of the samples were obtained with a UV–Vis–NIR spectrophotometer (UV-3600, Shimadzu, Kyoto, Japan) in the wavelength range of 200–2500 nm. The temperature and thermal images were captured using a FOTRIC 246M infrared camera (China).

### 2.5. Solar Light-Driven Evaporation of Water

As shown in Figure 1b, the solar interface evaporator was composed of thermal insulation foam (ethylene vinyl acetate (EVA) foam) and NTCP foam. The solar evaporator was held above the water and kept floating by using EVA foam, which prevented heat transfer to the water. Water was transported from the bottom up through the foam made of PU. A 300 W xenon lamp (GEL-S500, China Education Au-light Co., Ltd., Beijing, China) was used as a simulated solar light source, and an optical power density meter (CEL-NP2000, China Education Au-light Co., Ltd., Beijing, China) was used to correct the optical power density. The reactor was placed on an electronic balance, and the readings of the electronic balance were recorded at regular intervals. During the test, the infrared thermal imager (FOTRIC 246M) was used to record the surface temperature changes.

### 2.6. Photocatalytic Degradation Experiment

For this experiment, 10 mg/L rhodamine B (RhB) and methylene blue (MB) were prepared as target pollutants. The photocatalytic degradation performance was investigated via the same solar evaporation device used during solar evaporation. Before the photocatalytic performance test, the device was placed in the dark for 20 min. Samples were taken at regular intervals and measured by an ultraviolet–visible spectrophotometer during the experiment.

## 3. Results

### 3.1. Characterization of N-TiO_2_/C

**Figure 2:**
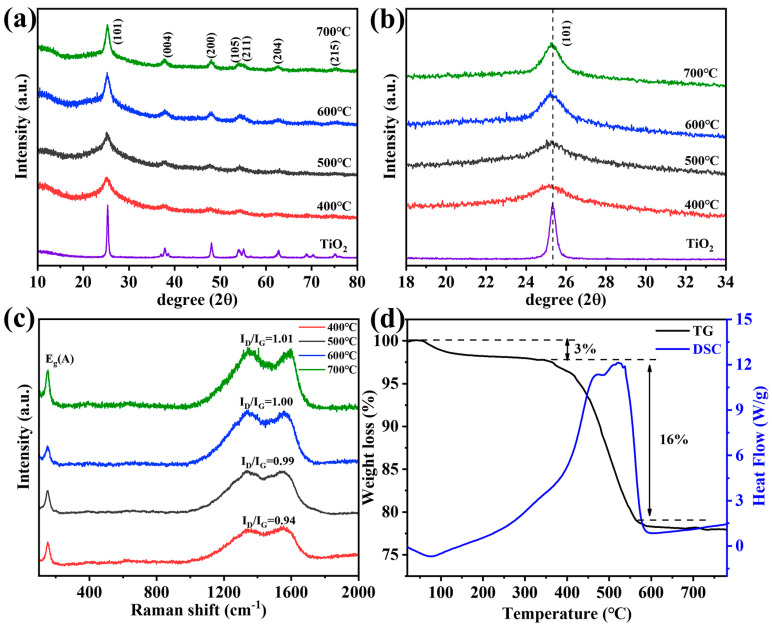
(**a**) XRD patterns, (**b**) XRD patterns in the 2θ range of 18–34°, (**c**) Raman spectrum, and (**d**) TGA curves of N-TiO_2_/C annealed at 400 °C.

We prepared a series of samples at different calcination temperatures (see the details in Supporting Information) and studied the crystal structure by XRD, as shown in Figure 2a. The diffraction peaks were well-indexed for the anatase TiO_2_ phase (PDF #21-1272) [33]. The crystallite size and interplanar distance of N-TiO_2_/C are listed in Table S1 and Note S1, Supporting Information. It is noteworthy that with the decrease in calcination temperature, the (101) lattice plane gradually shifted to the low-angle region (Figure 2b). The reason was that the ion radius of N^3−^ (0.146 nm) is larger than that of O^2−^ (0.14 nm), and according to the Bragg equation, N doping can cause lattice expansion [34]. N-doping can broaden the visible light response region, so the samples we prepared had relatively good photocatalytic activity. Raman spectroscopy was applied to further analyze the composition and structure of the N-TiO_2_/C, as shown in Figure 2c. The vibration band at 150 cm^−1^ (E_g_) was clearly observed, corresponding to anatase TiO_2_ [35]. In addition, we observed two peaks at about 1336 cm^−1^ and 1556 cm^−1^, which represent the D band and G band of carbon, respectively [36]. The carbon in N-TiO_2_/C gave a black color to the sample, which resulted from the calcination treatment of the HMT. The values of I_D_/I_G_ indicate the graphitization degree, and the higher the I_D_/I_G_ value, the lower the graphitization degree [37]. The sample annealed at 400 °C had the highest I_D_/I_G_ value, indicating a higher degree of graphitization. C with high graphitized carbon content can make electrons undergo transitions more easily and inhibit electronic recombination [38], thereby enhancing the light absorption ability in the near-infrared region. Therefore, the condition of 400 degrees was the preferred option. Thermogravimetric analysis was carried out from 20 °C to 800 °C at a heating rate of 10 °C min^−1^ in an air atmosphere. As can be seen in Figure 2d, the loss of 3% that occurred below 340 °C could be attributed to the evaporation of the adsorbed moisture or gaseous molecules. However, major weight loss (16%) took place in between 340 and 560 °C in the N-TiO_2_/C-400 sample, which was most likely the result of the loss of the carbon species in the material. The blue curve indicates that this part was mainly due to the heat released from carbon combustion.

**Figure 3:**
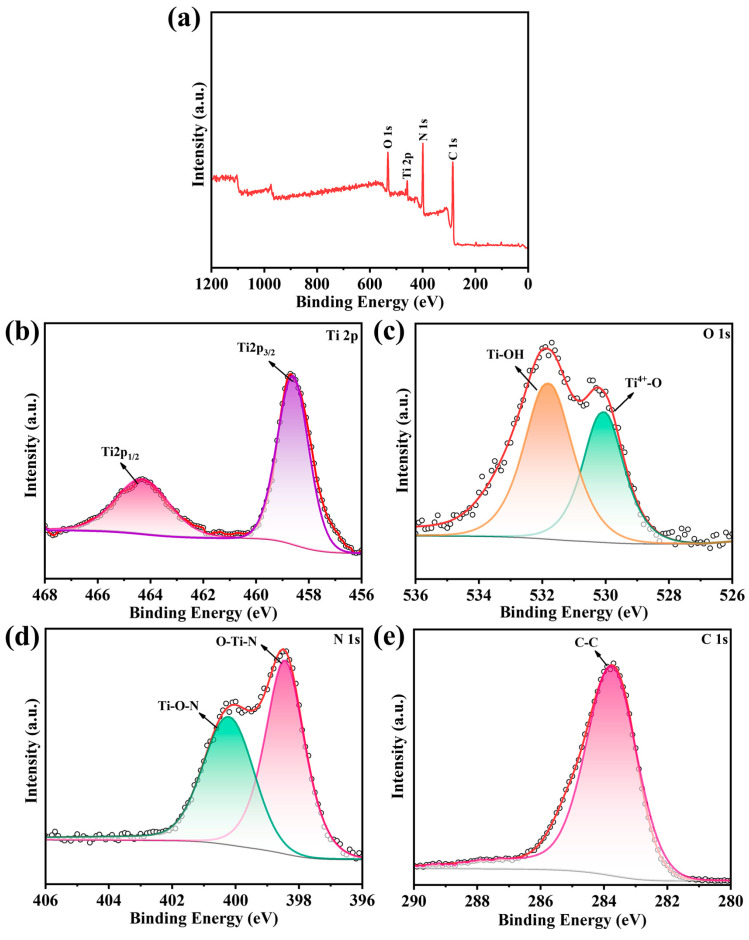
XPS spectra of the N-TiO_2_/C annealed at 400 °C. (**a**) The surveyed spectrum, with high-resolution spectra of (**b**) Ti 2p, (**c**) N 1s, (**d**) C 1s, and (**e**) O 1s.

The XPS spectra survey was carried out to analyze the composition and chemical state of the elements in the N-TiO_2_/C-400. In Figure 3a, the N-TiO_2_/C was mainly composed of Ti, N, C, and O elements. In Figure 3b, the peaks at 458.6 eV and 464.3 eV corresponded to the Ti 2p_3/2_ and Ti 2p_1/2_ peaks, respectively [39]. As shown in Figure 3c, the high-resolution XPS spectrum of O 1s had two peaks at 530.1 eV and 531.8 eV, which corresponded to Ti^4+^-O and Ti-OH bonds, respectively. The two peaks located at 398.4 eV and 400.2 eV (Figure 3d) were assigned for O-Ti-N and Ti-O-N bonds, respectively [40]. For the C 1s spectrum shown in Figure 3e, the binding energies at 284.4 eV were assigned for C-C bonds. The above results show that the N-TiO_2_/C material was successfully prepared by the one-step calcination method.

**Figure 4:**
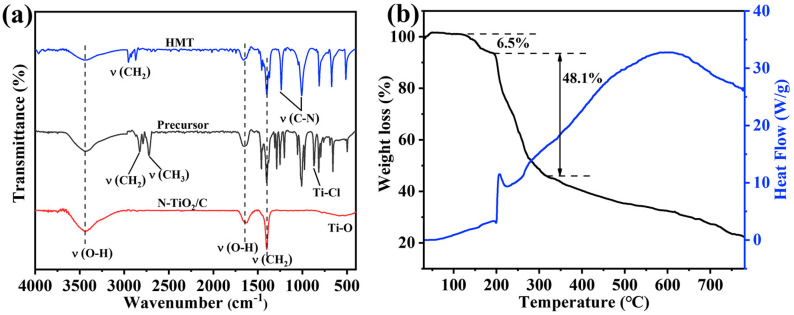
(**a**) FT-IR spectra of HMT, the precursor, and N-TiO_2_/C annealed at 400 °C; (**b**) TGA curves of the precursor of N-TiO_2_/C-400.

**Figure 5:**
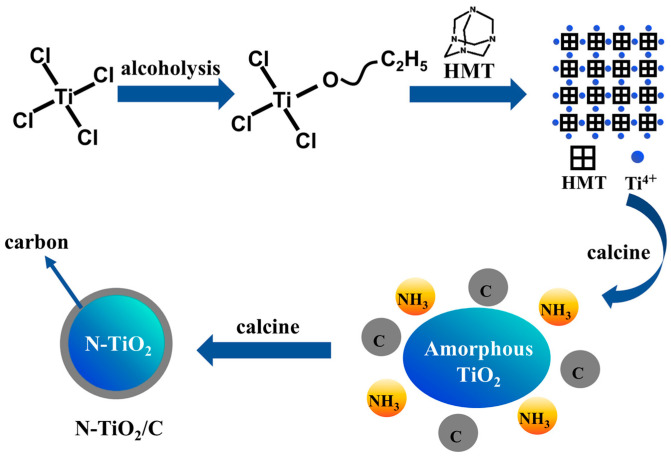
Schematic illustration of N-TiO_2_/C synthesis.

To investigate the formation mechanism, we conducted FT-IR and TGA tests, and the results are shown in Figure 4. The peaks at 3452 and 1652 cm^−1^ were attributed to the O–H stretching and the O–H bending vibrations [41]. The peaks at 2827, 2783, and 867 cm^−1^ were attributed to –CH_2_, –CH_3_, and Ti-Cl groups in the TiCl_x_(OC_2_H_5_)_4−x_ species, implying that TiCl_4_ participated in the alcoholysis reaction [42]. The peaks around 2900 cm^−1^ for HMT were assigned to the stretching vibrations of the -CH_2_ groups. The peaks at 1014 and 1242 cm^−1^ in HMT corresponded to the stretching vibrations of C-N bonds, and these peaks split into three peaks in the precursor. According to previous reports, this indicates that Ti-HMT coordination complexes were formed [43,44]. After adding HMT to this system, HMT molecules were coordinated to titanium, and the chloride anions were in the outer coordination sphere. A broad absorption band peak at 400–800 cm^−1^ in C/N-TiO_2_ could be the characteristic peaks of Ti-O of TiO_2_. Based on the above results, the generation process of the precursor can be described as followings. TiCl_4_ reacted with ethanol to form TiCl_x_(OC_2_H_5_)_4−x_, and then the metal–HMT complex precursor Ti(HMT)_2_Cl_x_(OC_2_H_5_)_4−x_ was obtained after mixing with HMT. Noticeably, this structure took full advantage of the high N content of HMT to prepare N-TiO_2_/C, and the doping level and N configuration were effectively modulated by controlling the annealing temperature and time. The formation mechanism was further analyzed based on the thermogravimetric image of the precursor (Figure 4b). The weight loss region from 128 °C to 200 °C resulted from the decomposition of hydroxyl and organic components from the precursor. The blue curve mainly represents the decomposition of precursors and the exothermic combustion reactions of organic compounds. The exothermic peak at around 250 °C corresponded to the combustion reaction of organic matter, releasing a large amount of heat. When the temperature rose to 320 °C, the weight decreased significantly, mainly due to the reaction of HMT in the precursor and the release of NH_3_. At this time, amorphous TiO_2_ released heat and transformed into anatase TiO_2_. Afterwards, the weight slowly decreased, mainly due to the formation process of N-TiO_2_ and the combination with carbon. The equation and schematic diagram (Figure 5) of the formation mechanism were as follows:

TiCl_4_ + C_2_H_5_OH → TiCl_x_(OC_2_H_5_)_4−x_ + HCl → Ti (HMT)_2_Cl_x_(OC_2_H_5_)_4−x_ (1)

HMT → C + NH_3_ (2)

Ti (HMT)_2_Cl_x_(OC_2_H_5_)_4−x_ + NH_3_ → N-TiO_2_ (3)

N-TiO_2_ + C → N-TiO_2_/C (4)

**Figure 6:**
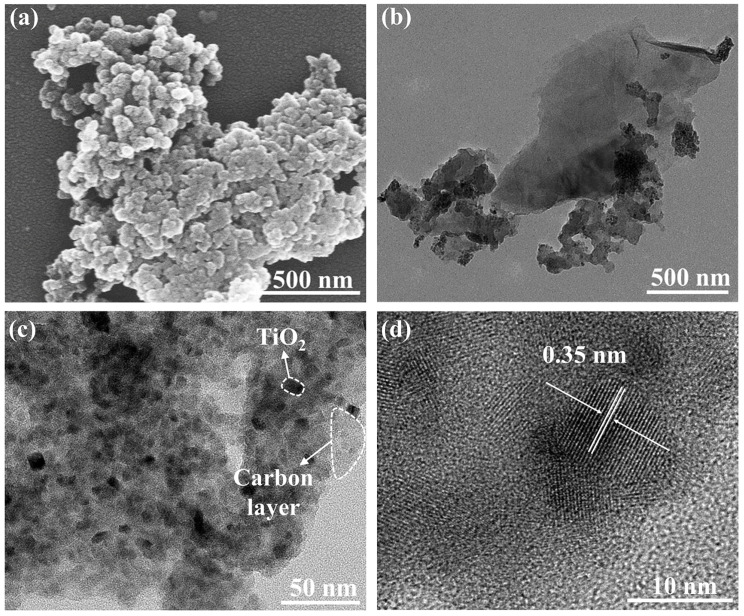
(**a**) SEM images, (**b**,**c**) TEM images, and (**d**) HRTEM images of N-TiO_2_/C annealed at 400 °C.

The microstructures of the sample annealed at 400 °C were studied by SEM and TEM. The SEM results (Figure 6a) showed that the sample formed a spherical morphology. The TEM images (Figure 6b) showed that the N-TiO_2_ nanoparticles were closely connected with the surrounding lamellar carbon. In addition to the surface carbon layer, carbon also appeared in the interstitial sites of the nanoparticles so that these nanoparticles seemed to be bonded together by carbon (Figure 6c). According to the HRTEM image (Figure 6d), the nanoparticles had good crystallinity and clear lattice stripes. The measured lattice spacing was 0.35 nm, which corresponded to the (101) plane of anatase TiO_2_. Particle size distribution was analyzed by the Nano Measurer 1.2 software, and 50 markers were selected from Figure 6c. In Figure S1 (Supporting Information), the particle size of the TiO_2_ nanoparticles was about 10.1 nm, which was close to the XRD calculation results.

### 3.2. Photothermal Conversion of N-TiO_2_/C

**Figure 7:**
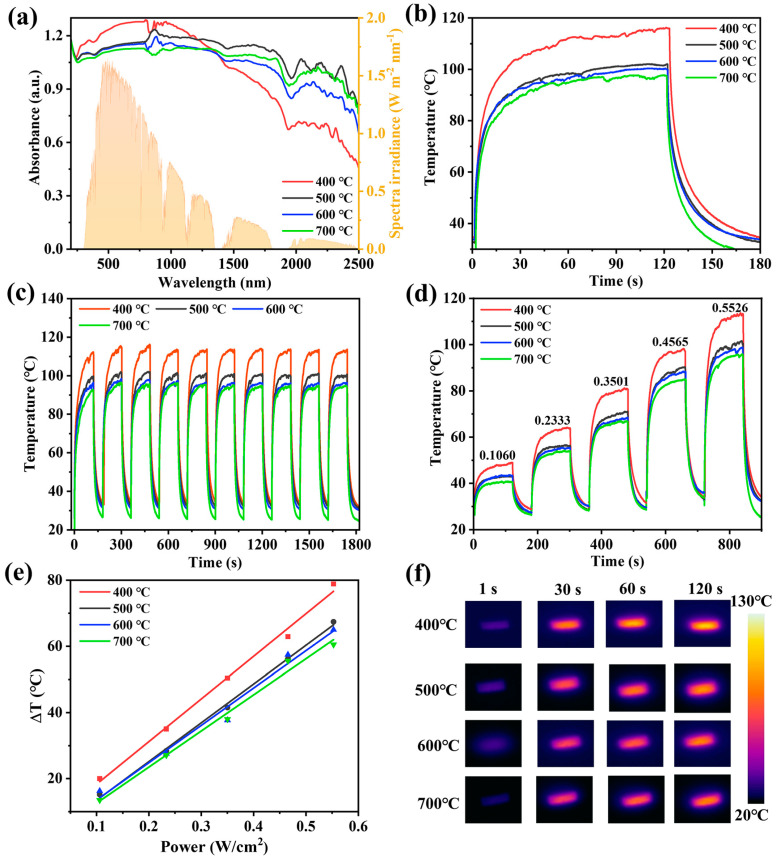
Effect of calcination temperature on photothermal properties of N-TiO_2_/C. (**a**) UV–Vis–NIR absorption spectra and solar spectrum (AM1.5G), (**b**) photothermal conversion curves, (**c**) stability test, (**d**) temperature changes at different NIR laser intensities, (**e**) linear relationship between temperature change and laser intensities, and (**f**) thermal images.

The UV–Vis–NIR absorption of samples at different calcination temperatures was analyzed. As shown in Figure 7a, N-TiO_2_/C has broad absorption in the visible–near-infrared regions. The yellow part in the figure shows the distribution of solar radiation energy (AM1.5G). In this system, carbon plays a role in broadening the absorption range of materials from visible light to near-infrared regions. Meanwhile, it can effectively utilize the absorbed light and convert it into thermal energy. It is noteworthy that the absorbance of N-TiO_2_/C-400 increased with longer wavelengths at 400–1000 nm, which means that the sample had a better absorption ability in the wavelength region with a high solar energy. The samples prepared at a low temperature had the highest nitrogen doping content, and thus had the smallest grain size, which is more conducive to light absorption. Through the Kubelka Mnik function (Note S2, Supporting Information), the band gaps of N-TiO_2_/C were found to be 2.33, 2.56, 2.58, and 2.93 eV (Figure S2, Supporting Information). It can be seen that with the increase in temperature, the optical band gap increased significantly. Some localized N 2p states were formed above the valence band edge in N-doped TiO_2_. The energy of excitation from the impurity states of N 2p to the conduction band was reduced [45]. Compared with the wide band gap of TiO_2_ (3.2 eV), the narrow band gap of N-TiO_2_/C is more conducive to broadening the absorption range of visible light and simultaneously promotes the separation of photogenerated electrons and holes.

We used infrared thermal imaging to monitor the photothermal effect of N-TiO_2_/C. It was found that as soon as the 808 nm irradiation laser was turned on, the surface temperature rose sharply, indicating that N-TiO_2_/C has a fast optical response ability. After irradiation for 120 s, the surface temperature tended to be stable, and the equilibrium temperatures of N-TiO_2_/C-x (x = 400, 500, 600, and 700) reached 116 °C, 101.5 °C, 100.2 °C, and 97.8 °C, respectively (Figure 7b). Moreover, after ten heating–cooling cycles, the temperatures of the samples maintained a stable heating rate and reached the highest temperature, confirming the superior thermal stability of N-TiO_2_/C (Figure 7c). Meanwhile, the temperature increments (ΔT) of all samples exhibited a power density-dependent photothermal effect at different excitation powers (Figure 7d,e), suggesting that the photothermal conversion behavior of N-TiO_2_/C can be adjusted by changing the excitation power. Among them, N-TiO_2_/C-400 had the best photothermal performance, which was attributed to the maximum degree of graphitization. With more sp^2^ hybridized carbon atoms, the light energy matched by the excited electron transition was smaller, thereby enhancing the light absorption ability of N-TiO_2_/C-400 in the near-infrared region. Figure 7f demonstrates the photothermal imaging capacity more intuitively. According to its cooling curve (Note S3, Figures S3–S6, and Table S2, Supporting Information), the photothermal conversion efficiencies of N-TiO_2_/C-400, N-TiO_2_/C-500, N-TiO_2_/C-600, and N-TiO_2_/C-700 were calculated to be 48.2%, 39.0%, 35.3%, and 31.3%, respectively. Therefore, 400 °C was selected as the optimal calcination temperature, and an interfacial water evaporation system was established with porous polyurethane (PU) foam.

### 3.3. Water Evaporation Performance of NTCP

**Figure 8:**
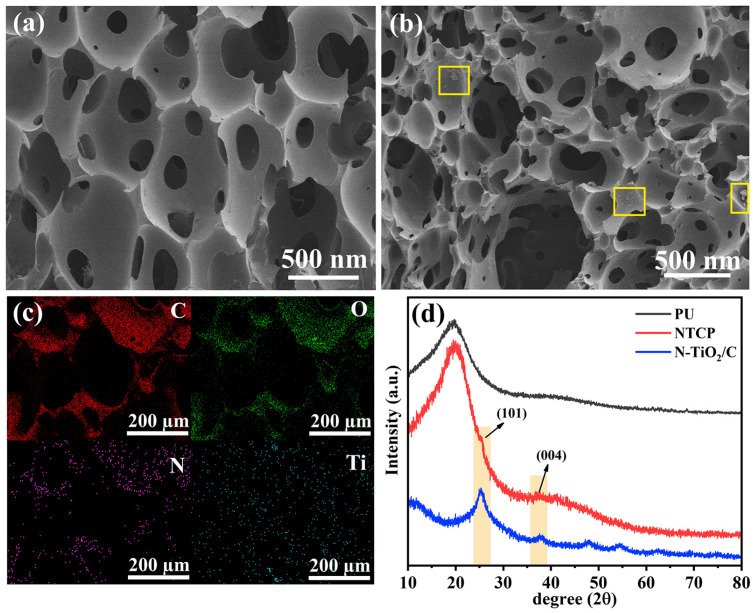
(**a**) SEM image of PU; (**b**) SEM image of NTCP; (**c**) mapping images of NTCP; and (**d**) XRD patterns of N-TiO_2_/C, PU, and NTCP.

We combined photothermal materials with polyurethane to prepare a foam-like evaporation device, N-TiO_2_/C/PU (NTCP). We measured SEM images and XRD patterns of NTCP. NTCP foam had a rougher surface and more pores than PU foam, indicating that N-TiO_2_/C was effectively doped into the PU frame (Figure 8a,b). In addition, the combination of N-TiO_2_/C and PU increased its porosity, which is conducive to light absorption and water transport. The mapping images of the NTCP foam surface also prove this point, from which C, O, N, and Ti are evenly distributed on the foam skeleton (Figure 8c). The XRD pattern (Figure 8d) demonstrated that N-TiO_2_/C kept its crystal structure well after being doped into PU foam. In the liquid flow experiment, the tissue on the NTCP foam soaked in blue liquid turned blue quickly (Figure S7, Supporting Information). This confirmed that the NTCP foam had a high water delivery efficiency.

To investigate the effect of the amount of N-TiO_2_/C on the optical properties of NTCP foam, 10, 30, 50, and 100 mg N-TiO_2_/C were added to form foams. Compared with pure PU foam, the absorbance spectra of NTCP foam showed a broad absorption covering almost the full solar adsorption spectrum (Figure S8a, Supporting Information). When the content of N-TiO_2_/C increased, the absorption spectrum of NTCP foam was strengthened and broadened. Under the simulated light intensity of 1 kW m^−2^, the equilibrium temperature of NTCP-100 mg reached the highest temperature (72.5 °C) within 240 s, while pure PU foam only reached 37 °C (Figure S8b, Supporting Information). This indicated that N-TiO_2_/C is a good photothermal material. Figure S8c (Supporting Information) exhibited a superior anti-photobleaching property after five heating–cooling processes. The real-time temperature changes of NTCP foams in the water evaporation experiments were monitored by infrared camera (Figure S8d, Supporting Information). For subsequent experiments, 100 mg of photothermal powder was used as the condition.

**Figure 9:**
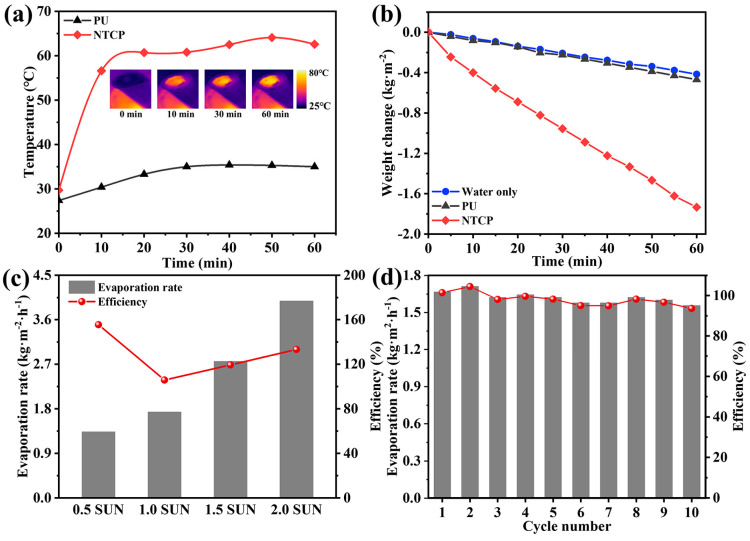
(**a**) The temperature changes of two foams floating on water against irradiation time; inserted figure: surface temperature of NTCP during water evaporation experiment. (**b**) Water evaporation curves, (**c**) solar vapor generation performance at different simulated solar illumination powers, and (**d**) stability test.

We designed a set of testing devices to record the change in water quality during solar-driven water evaporation. The real-time temperature changes of NTCP foams in the water evaporation experiments were monitored by an infrared camera. In Figure 9a, it is found that NTCP was accompanied by a significant temperature rise, and the final temperature reached 62 °C. In contrast, the temperature of PU only changed slightly, and its final temperature was 35 °C. This proves that the heat energy converted from sunlight can be exactly located in the NTCP foam. To evaluate the efficiency of water evaporation, the mass change in water was recorded during the irradiation process (Figure 9b). The calculated evaporation rate and solar–vapor conversion efficiency are listed in Note S4 and Table S3, Supporting Information [46,47]. NTCP foam significantly accelerated the rate of water evaporation, and the evaporation rate of NTCP foam was 1.73 kg m^−2^ h^−1^, which was prominently higher than that of individual water (0.41 kg m^−2^ h^−1^) and PU foam (0.47 kg m^−2^ h^−1^). The solar interfacial evaporation performance of the NTCP evaporator was further investigated under a series of simulated solar irradiation intensities. As shown in Figure 9c, when the solar illumination was increased to 0.5, 1, 1.5, and 2 sun (kW m^−2^), the surface temperature of NTCP also increased, and the evaporation rate gradually increased to 1.33, 1.73, 2.76, and 3.98 kg m^−2^h^−1^, respectively. Consequently, the evaporation efficiency was 155.5, 105.8, 119.3, and 133.2%, respectively. To verify the stability of photothermal conversion materials, the NTCP foam was placed onto the water and irradiated in simulated sunlight of 1 kW m^−2^ for 1 h each time for 10 cycles (Figure 9d). Therefore, NTCP foam can maintain stable photothermal performance for a long time, which is of great significance to its practical application. Table S4 (Supporting Information) displays the water evaporation properties of reported photothermal materials under 1 sun illumination [48,49,50,51,52,53]. It can be seen that the water evaporation rate and efficiency of the NTCP evaporator in our work were higher than most evaporators based on foam, indicating that NTCP is a promising candidate for high-efficiency solar evaporation in practical applications.

We also built an outdoor solar evaporation device, which was mainly composed of an evaporating chamber, NTCP, and water collecting tank (Figure S9a,b, Supporting Information). The vapor condensate water gathered in the bottom along the slope and was collected through the water outlet. The solar light intensity gradually increased from 9:00 a.m. to 12:00 a.m. and reached its maximum (838 W m^−2^) at 12:00 a.m. (Figure S9c, Supporting Information). Accordingly, the evaporation rate of the evaporator increased from 0.3259 kg m^−2^ h^−1^ to 0.8814 kg m^−2^ h^−1^ (Figure S9d, Supporting Information). From 12:00 a.m. to 2:00 p.m., the solar light intensity decreased slightly. The device was placed outdoors for a 6 h evaporation test, and water mist appeared on the condensation cover (Figure S10, Supporting Information). At this time, the evaporator rate did not change much (0.7481 to 0.8814 kg m^−2^ h^−1^). The above results prove that the NTCP evaporator also had good evaporation performance under natural light.

**Figure 10:**
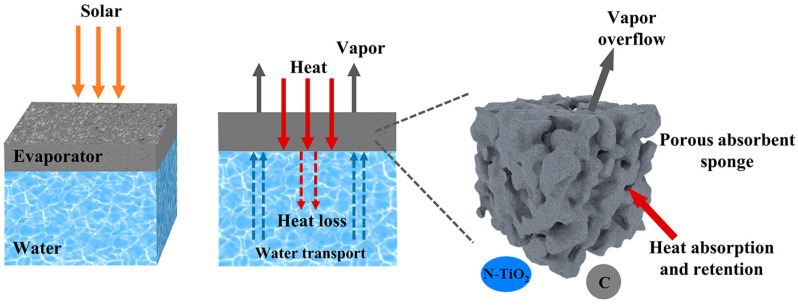
The schematic for the solar interfacial evaporation mechanism of NTCP.

The schematic diagram of the solar interfacial evaporation mechanism is shown in Figure 10. The excellent water evaporation performance of NTCP foam is the result of multiple synergistic effects: Firstly, N doping and carbon modification improved the light absorption of TiO_2_. Then, the light-induced effect caused by carbon converted light into heat. Secondly, the porosity of PU foam ensured a stable water supply source in the process of water evaporation. At the same time, the porous structure facilitated the overflow of vapor. Thirdly, the absorbed heat remained on the foam without being dispersed into the bulk water.

### 3.4. Photodegradable Organic Dye Properties of NTCP

**Figure 11:**
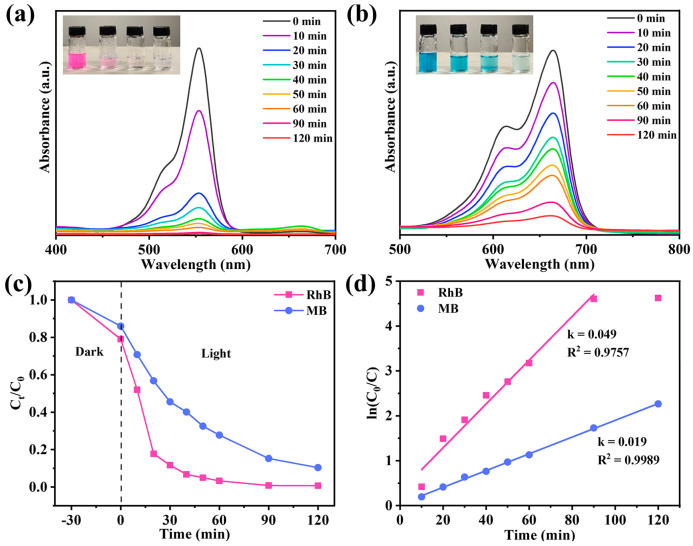
(**a**,**b**) Degradation absorbance spectra of RhB and MB; insert figures: recording of solutions every 20 min during photocatalytic degradation process. (**c**) RhB and MB photodegradation over NTCP foam and (**d**) reaction kinetics of RhB and MB photodegradation.

In the actual environment, most water contains harmful organic pollutants, such as dyes. These pollutants may pollute the photothermal evaporator and affect its evaporation efficiency during long-term operation. Therefore, our evaporation system should have photodegradation performance to ensure the stable operation of the system. TiO_2_ has been well known as an efficient photocatalyst for organic pollutants [54]. In this study, NTCP-100 mg was selected for photodegradation of methylene blue (MB) and rhodamine B (RhB). As depicted in Figure 11a,b, the absorption peaks of RhB and MB gradually disappeared with illumination time. The removal rates of RhB and MB reached 99.9% and 90.6%, respectively (Figure 11c). Moreover, RhB and MB degradation kinetics based on pseudo-first order (Figure 11d) fit the experimental data under irradiation of visible light, giving 0.049 and 0.019 min^−1^ of a rate constant (k value), respectively. This further supports the excellent photocatalytic performance of NTCP for dye photodegradation under irradiation of visible light. The excellent visible light photocatalysis of NTCP is due to the unique adsorption of visible light to excite electrons from its new state to its conduction band. In addition, the water evaporation rates of NTCP in RhB and MB solutions were 1.61 and 1.60 kg m^−2^ h^−1^, respectively, which were close to those in pure water (Figure S11, Supporting Information).

**Figure 12:**
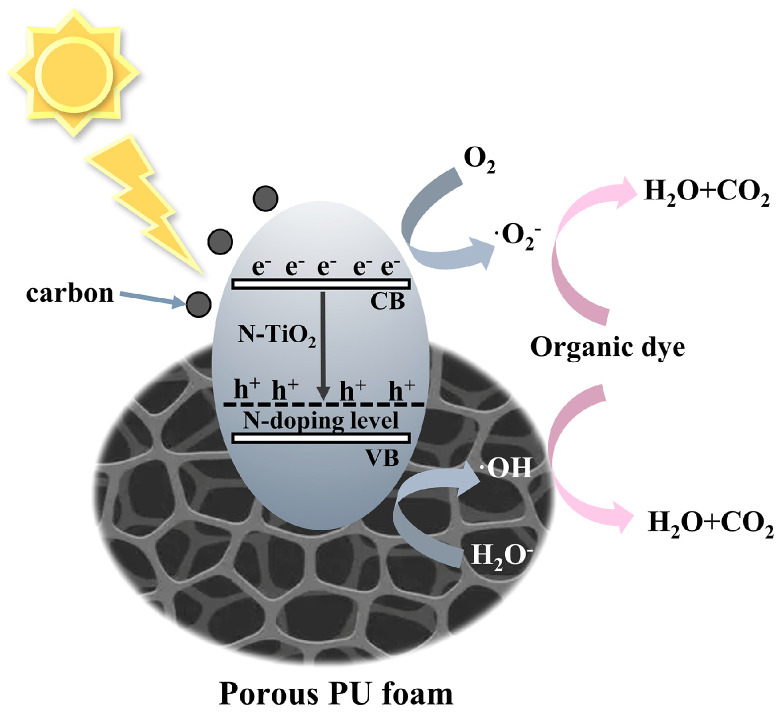
Photothermal synergetic photocatalytic mechanism of NTCP foam.

To determine the main active components, reactive oxygen species capture experiments were conducted. In Figure S12 (Supporting Information), ascorbic acid (L-AA), isopropanol (IPA), and triethanolamine (TEOA) were used as ·O^2−^, ·OH, and h^+^ scavengers, respectively. The addition of L-AA and IPA inhibited the degradation of RhB, indicating that photogenerated ·O^2−^ and·OH are the main active substances in C/N-TiO_2_ photocatalysis. Based on the above analysis, we proposed the mechanism of photodegradation by NTCP foam (Figure 12). In the NTCP composite, the PU foam fulfills the role of adsorption and the fixed load of C/N−TiO_2_, providing more active surface sites for light adsorption and photocatalysis. The element N was doped into the TiO_2_ lattice, resulting in an N-doping level above the valence band of TiO_2_ to narrow the band gap of TiO_2_. Additionally, carbon enhanced the absorption ability and promoted the separation of the photogenerated electrons and holes. When solar light with a bandgap greater than 2.33 eV was absorbed by C/N-TiO_2_ particles, the electrons in their valence band (VB) were excited to the conduction band (CB), forming electron–hole pairs and undergoing redox reactions, which can catalyze the photocatalytic degradation of organic pollutants [55].

## 4. Conclusions

In summary, we reported N-TiO_2_/C composites with a broad absorption range and good photothermal conversion performance prepared by the sol-gel method. The N doping level can be effectively modulated by controlling the annealing temperature in the presence of hexamethylenetetramine as a source of nitrogen and carbon. The sample prepared at 400 °C demonstrated the best photothermal performance for the graphitization degree of C, reducing the energy required for electron transition, and thus having better optical properties in the near-infrared spectrum. Additionally, N-TiO_2_/C-400 had a smaller grain size, which is favorable for light absorption. Therefore, N-TiO_2_/C-400 achieved the optimal photothermal performance with a photothermal efficiency of 48.2%. Meanwhile, N-doping narrowed the band gap and broadened the absorption range to the visible light region, thereby enhancing the photocatalytic activity. The photothermal foam evaporator prepared with the photothermal N-TiO_2_/C powder exhibited an evaporation rate of 1.73 kg m^−2^h^−1^ and a solar–vapor conversion efficiency of 105.8% under the irradiation of 1 kW m^−2^. The outdoor experiment proves that the NTCP-based solar evaporation system can obtain 4.07 kg m^−2^ of freshwater in the daytime (9:00 a.m. to 2:00 p.m.), giving it great potential for practical application. In addition, the evaporator had good cycling stability and self-cleaning capability to resist harsh water environments, such as organic contamination conditions. In the photodegradation experiments, the removal rates of RhB and MB reached 99.9% and 90.6%, respectively. Overall, research on the N-TiO_2_/C/PU can maximize the utilization of light energy and will be a promising strategy to meet the demand for fresh water.

## Data Availability

The original contributions presented in this study are included in the article/Supplementary Material. Further inquiries can be directed to the corresponding author(s).

## Supplementary Materials

The following supporting information can be downloaded at: https://www.mdpi.com/article/10.3390/ma18071550/s1, Table S1: Structural characteristic of N-TiO_2_/C. Note S1: Scherrer equation and Bragg equation. Note S2: The calculation of optical bandgap. Figure S1: Average particle size distribution of N-TiO_2_/C-400. Figure S2: Band gap spectra of sample N-TiO_2_/C. Note S3: The method for measuring the photothermal conversion efficiency. Table S2: Efficiency of photothermal conversion of N-TiO_2_/C. Figures S3–S6: The cooling curve of N-TiO_2_/C and its corresponding time-lnθ linear curve. Figure S7: Water transport ability of NTCP foam. Figure S8: Photothermal properties of NTCP with different loads. Note S4: The calculation of evaporation rate and solar-vapor conversion efficiency. Table S3: The calculation results of solar-vapor conversion efficiency of all samples. Table S4: Comparison of the steam generation rate and efficiency based on foam in this work with that reported in previous literatures under one sun irradiation. Figure S9: Outdoor evaporation experiments results. Figure S10: Evaporation process of outdoor evaporation device. Figure S11: The water mass changes of NTCP in RhB and MB solutions. Figure S12: Photodegradation efficiencies in the presence of different quenchers.
