# Supplementary material for: Bifunctional N-TiO2/C/PU Foam for Interfacial Water Evaporation and Sewage Purification
Source: Materials (Basel). 2025 Mar 29;18(7):1550. doi: 10.3390/ma18071550 (PMC11990650; doi:10.3390/ma18071550)
Supplement: Supplementary file 1 [file materials-18-01550-s001.zip › materials-3532857-supplementary.pdf]

Supporting Information

Bifunctional N-TiO_2/C/PU Foam for Interfacial Water Evaporation and Sewage Purification

Ke Wang [1], Weifeng Li [1,*] and Yumei Long [1,2,*]

[1] College of Chemistry, Chemical Engineering and Materials Science, Soochow University, Suzhou 215123, China; kewang00203@163.com
[2] The Key Lab of Health Chemistry and Molecular Diagnosis of Suzhou, Soochow University,
Suzhou 215123, China
* Correspondence: liweifeng@suda.edu.cn (W.L.); yumeilong@suda.edu.cn (Y.L.)

Table S1. Structural characteristic of N-TiO_2/C.

Sample	2θ	FWHM	[a]Crystallite size (nm)	[b]Interplanar distance (nm)
N-TiO_2/C-400	25.13	0.82	10.0	0.352
N-TiO_2/C-500	25.24	0.80	10.4	0.352
N-TiO_2/C-600	25.25	0.69	11.4	0.352
N-TiO_2/C-700	25.30	0.68	12.5	0.353

[a] Determined by XRD using the Scherrer equation (Equation S1).
[b] Calculated from the Bragg equation (Equation S2).

Supplementary Note S1: Scherrer equation and Bragg equation:

$$d = k\lambda/\beta cos\theta \quad (1)$$

$$2dsin\theta = n\lambda \quad (2)$$

Where d (nm) is the grain size, and k (0.89) is the Scherrer constant. λ is the wavelength of the incident X-ray. θ and β are the Bragg diffraction angle and the full width at half maximum of the diffraction peak, respectively.

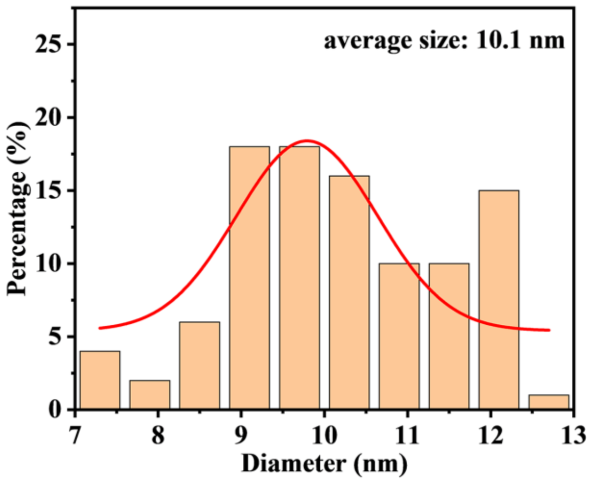

Figure S1. Average particle size distribution of N-TiO_2/C-400.

Supplementary Note S2: For the calculation of optical bandgap, Kubelka Mnik function is used in the range of 200-800 nm, and the equation is as follows:

$$\alpha h\nu = A\left(h\nu E_g\right)^{\frac{1}{2}} = A\left(h\frac{\lambda}{c} - E_g\right)^{\frac{1}{2}} \qquad (3)$$

Among them, α is the absorption coefficient, h (6.63×10^{-34} J) is the Planck constant, ν represents the frequency of light. A is a constant, and Eg (eV) is the bandgap width. λ (nm) and c (3×10^{8} m/s) are the wavelength and the speed of light, respectively.

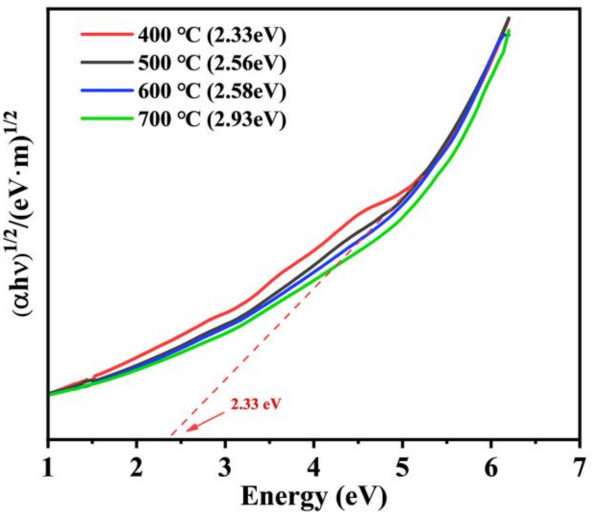

Figure S2. Band gap spectra of sample N-TiO_2/C.

Supplementary Note S3: The method for measuring the photothermal conversion efficiency of N-TiO_2/C is as follows: evenly spread the powder on quartz glass, irradiate it with 808 nm laser until the temperature reaches equilibrium, turn off the laser, and start recording the curve of time and temperature. The calculation process is as follows：

a) The total energy balance formula of the system:

$$\sum_i m_i C_{pi} \frac{dT}{dt} = Q_s - Q_{loss} \tag{4}$$

Among them, m_i (0.31g) and C_{pi} (0.8J/g ℃) represent the mass and heat capacity of the system (N-TiO_2/C and quartz glass), respectively. Because the mass of N-TiO_2/C is negligible compared with that of quartz glass, the heat capacity of quartz glass is used for calculation. Q_s is the photothermal heat energy input by the laser irradiation sample, and Q_{loss} represents the heat energy lost to the environment. When the temperature rises to the maximum, the system is in equilibrium, and $Q_s = Q_{loss} = hS\Delta T_{max}$. Where h is the heat transfer coefficient, S is the system area, and ΔT_{max} is the temperature difference between the maximum temperature and the ambient temperature.

b) Photothermal conversion efficiency:

The calculation results of s photothermal conversion efficiency of all samples were listed in Table S2.

$$\eta = hS\Delta T_{max}/(1 - 10^{-A_{808}}) \tag{5}$$

$$\tau_s = \sum_i m_i \, C_{P,i}/\text{hS} \tag{6}$$

$$\theta = \text{T} - T_{surr}/T_{max} - T_{surr} \tag{7}$$

$$d\theta/dt = Q_s/\tau_s hS\Delta T_{max} - \theta/\tau_s \tag{8}$$

Where I (0.4 W cm^{-2}) is the laser power and A_{808} (1.286) is the absorbance of the sample (N-TiO_2/C-400) at the wavelength of 808 nm. T is the real-time temperature of the sample, T_{max} (70 ℃) is the highest temperature of the cooling curve of the sample, and T_{surr} (28 ℃)is the ambient temperature. The laser is turned off, $Q_s = 0$. At this time, $d\theta/dt = -\theta/\tau_s$, $t = -\tau_s ln\theta$. According to Fig. S4, the value of τ_s is 56.9, and the photothermal conversion efficiency of N-TiO_2/C is 48.2%.

Table S2. Efficiency of photothermal conversion (η) of N-TiO_2/C

.Sample	T_{max} (°C)	T_{surr} (°C)	A_{808}	τ_s (s)	Efficiency of photothermal conversion (η)
N-TiO_2/C-400	70	28	1.286	56.9	48.2%
N-TiO_2/C-500	66	28	1.174	64.7	39.0%
N-TiO_2/C-600	65	28	1.133	70.1	35.3%
N-TiO_2/C-700	60	28	1.111	68.8	31.3%

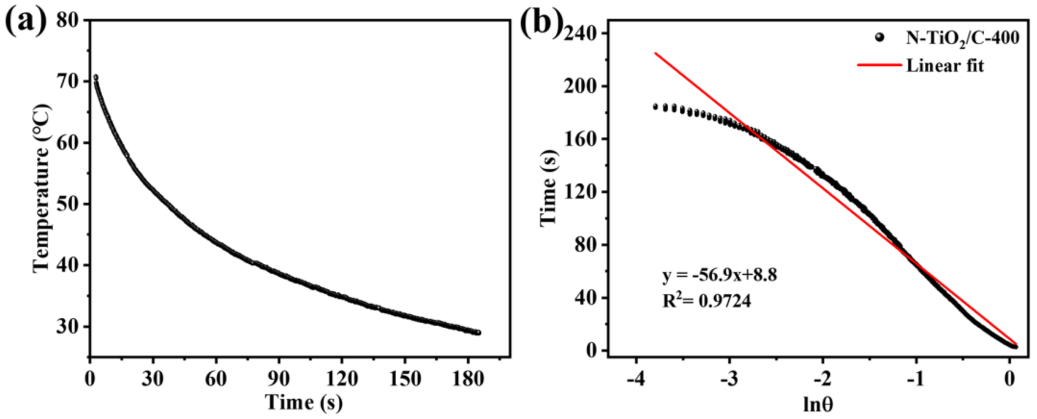

Figure S3. (a) The cooling curve of N-TiO_2/C-400 after the irradiation of laser, and (b) its corresponding time-lnθ linear curve.

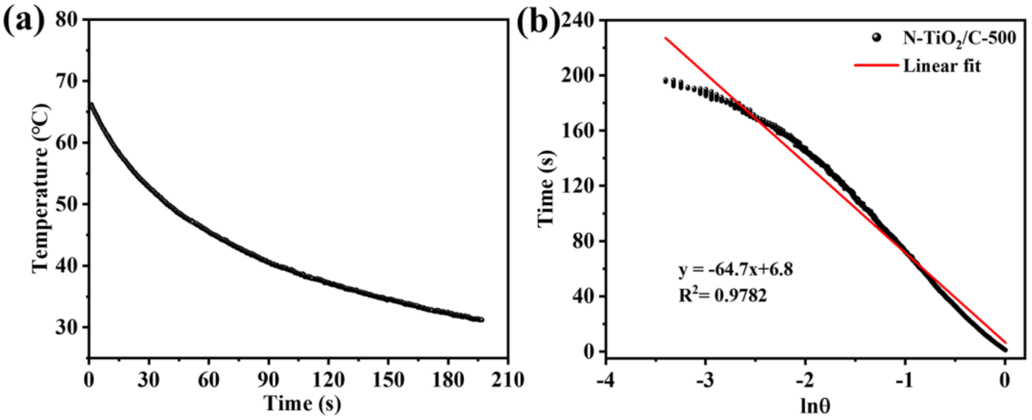

Figure S4. (a) The cooling curve of N-TiO_2/C-500 after the irradiation of laser, and (b) its corresponding time-lnθ linear curve.

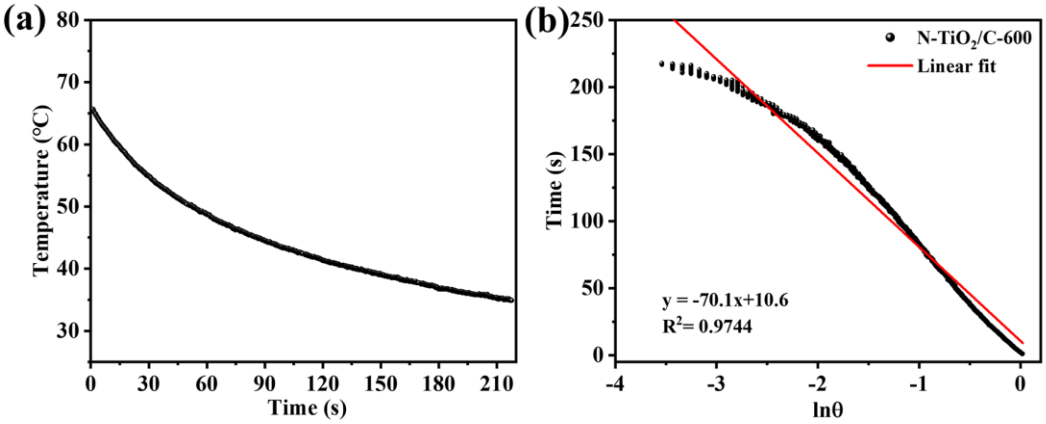

Figure S5. (a) The cooling curve of N-TiO_2/C-600 after the irradiation of laser, and (b) its corresponding time-lnθ linear curve.

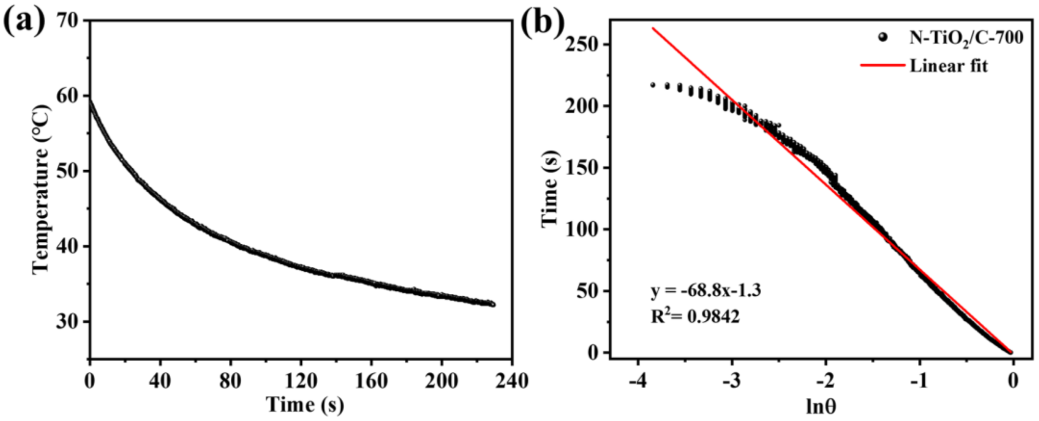

Figure S6. (a) The cooling curve of N-TiO_2/C-700 after the irradiation of laser, and (b) its corresponding time-lnθ linear curve.

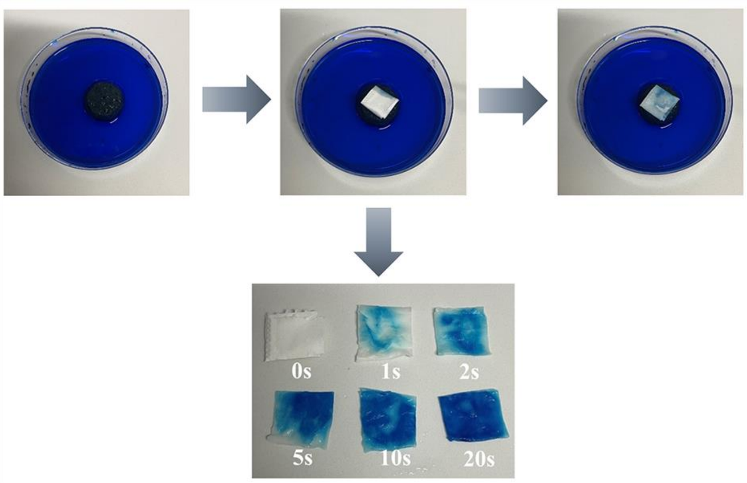

Figure S7. Water transport ability of NTCP foam.

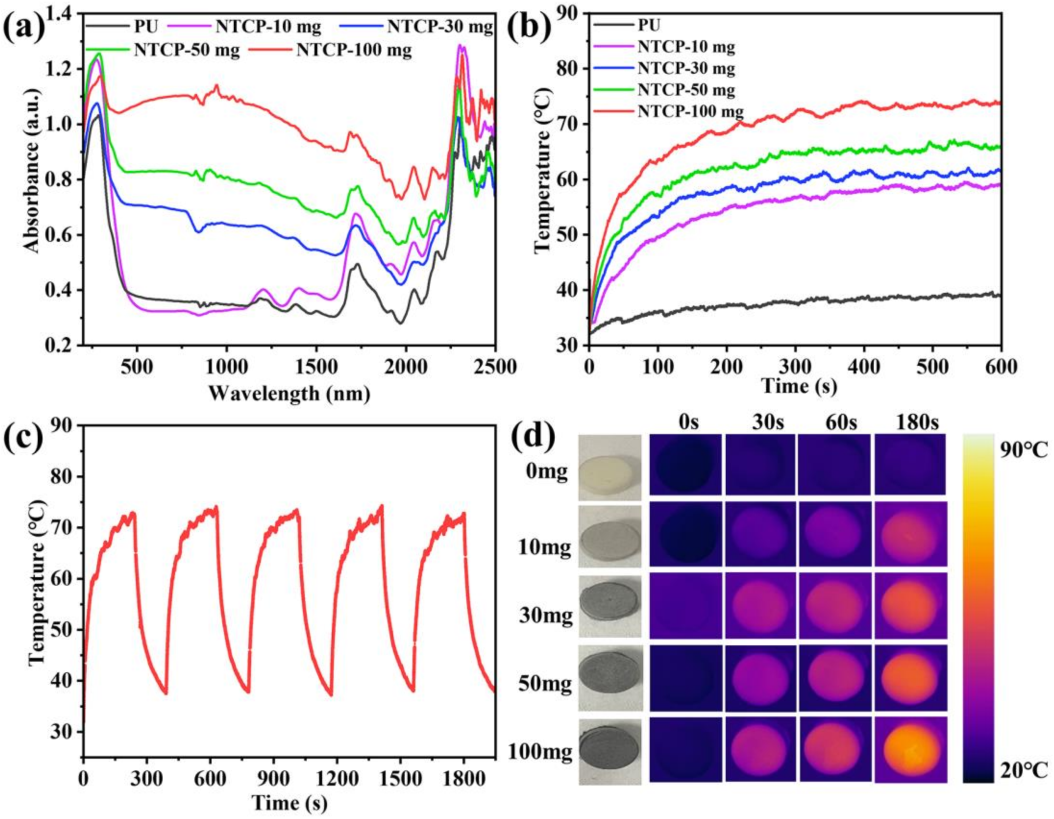

Figure S8. Photothermal properties of NTCP with different loads. (a) absorption spectra, (b) photothermal conversion curves under 1 sun illumination, (c) stability test of NTCP-100 mg, and (d) thermal images of NTCP.

Supplementary Note S4: The calculation evaporation rate and solar-vapor conversion efficiency:[1, 2]

a) Evaporation rate (ν):

$$\nu = \Delta m/S \times t \tag{9}$$

b) Evaporation efficiency (η):

The evaporation efficiency can be obtained as follows, and the related data was shown in Table S3.

$$\eta = (\Delta\nu \times (L_\nu + Q))/P_{in} \tag{10}$$

where Δm (kg), is the mass change of water, S (m^2) is the irradiation area of the upper surface of the photothermal conversion material, and t (h)is the irradiation time. $\Delta\nu$ (kg $m^{-2}h^{-1}$) is the evaporation rate under illumination conditions subtracted from that under dark conditions, L_ν is the latent heat of phase change of water, Q (kJ kg-1) denotes the sensible heat per unit mass of water, and P_{in} indicateds the input solar energy. $Q = c\times(T_2$

$-T_1)$, c is the specific heat capacity of water, T_1 is the initial temperature of water, and T_2 is the evaporation temperature.

Table S3. The calculation results of solar-vapor conversion efficiency of all samples.

**Material**	**Evaporation rate (kg m^{-2} h^{-1})**	**Dark evaporation rate (kg m^{-2} h^{-1})**	**T_1 (℃)**	**T_2 (℃)**	**Latent heat**	**Sensible heat**	**Conversion Efficiency (%)**
PU	0.46	0.15	27.4	35	2415	31.92	21.3
NTCP	1.73	0.2	29.7	62.6	2345.5	138.18	105.8
0.5 SUN	1.33	0.2	27.2	50.4	2372	97.44	155.5
1.5 SUN	2.75	0.2	27.4	71.5	2335.3	185.22	119.3
2 SUN	3.98	0.2	26.2	84.7	2293.1	245.7	133.2

Table S4. Comparison of the steam generation rate and efficiency based on foam in this work with that reported in previous literatures under one sun irradiation.

**Absorbing material**	**Steam generation efficiency (%)**	**Steam generation rate (kg m^{-2} h^{-1})**	**Refs.**
MoS_2@sponge	86.2	1.2	[3]
[a]PANI/HNTs@PU	94.7	1.61	[4]
[b]PI-CB-MHGM membrane	86.65	1.49	[5]
[c]SCF@wPU	67.7	1.1	[6]
polypyrrole/polyurethane composite foams	86.9	1.334	[7]
$Ti_3C_2T_x$/polyurethane	84.85	1.35	[8]
**NTCP**	**105.8**	**1.73**	**This work**

[a] polyaniline/halloysite nanotubes decorated PU nanofiber.

[b] polyimide-based porous membrane with modified hollow glass microspheres and carbon black.

[c] carbon fabric/polyurethane Janus membranes.

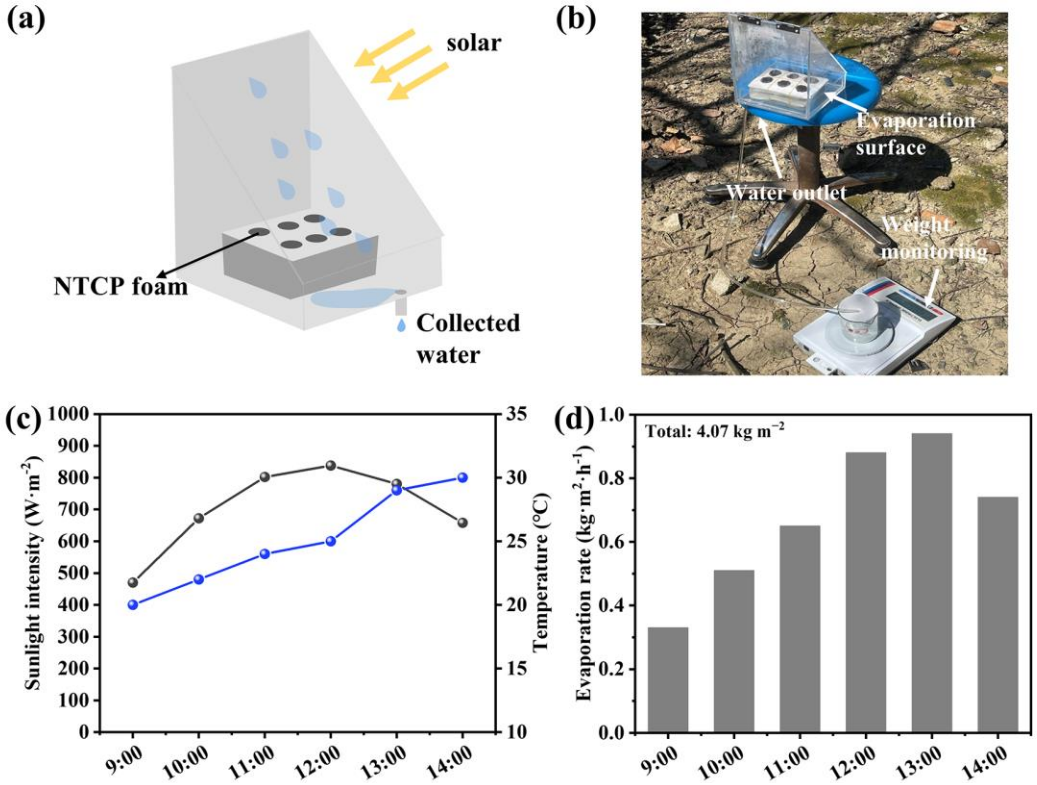

Figure S9. (a) Outdoor evaporation testing device, (b) photographs of device, (c) changes of solar light intensity and temperature, and (d) the change of evaporation rate.

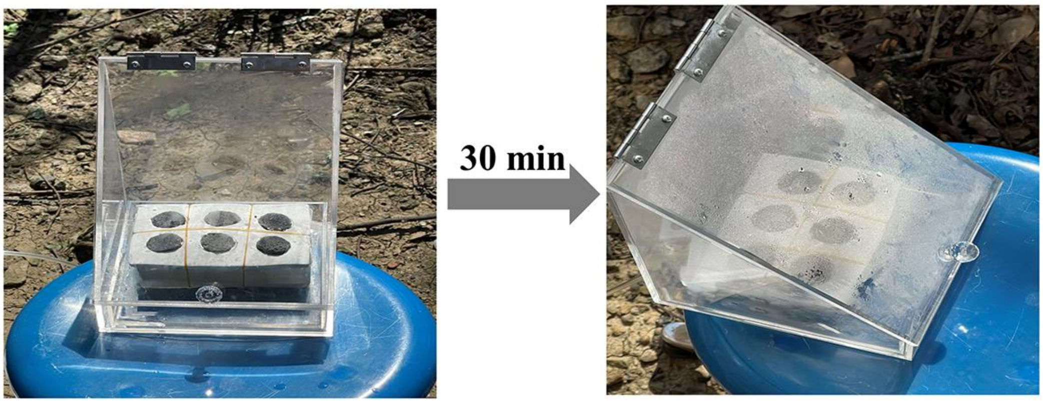

Figure S10. Evaporation process of outdoor evaporation device.

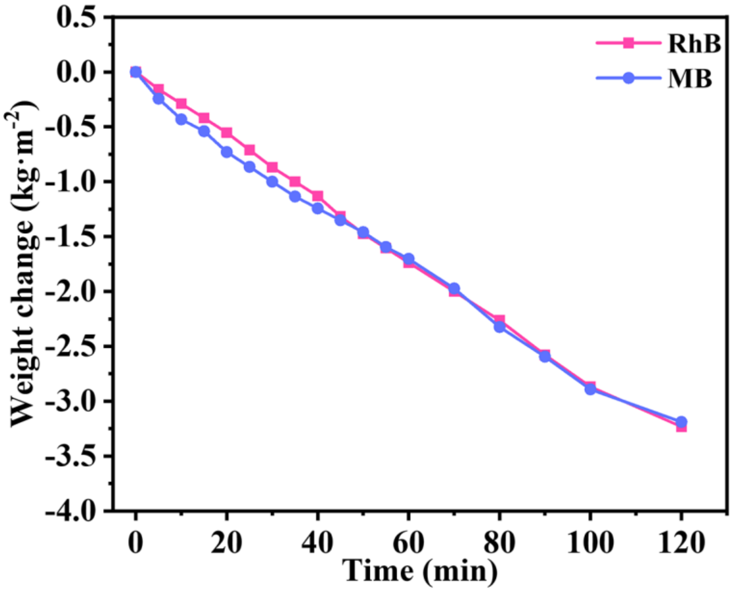

Figure S11. The water mass changes of NTCP in RhB and MB solutions.

In order to investigate the role of three active substances O^{2-}, $\cdot OH$, and h^+ in the photocatalytic process, 1mmol/L ascorbic acid (L-AA), isopropanol (IPA), and riethanolamine (TEOA) was added to 10 mg/L of RhB solution, respectively. Then, 20 mg of C/N-TiO_2 was added as a catalyst for photocatalytic experiments.

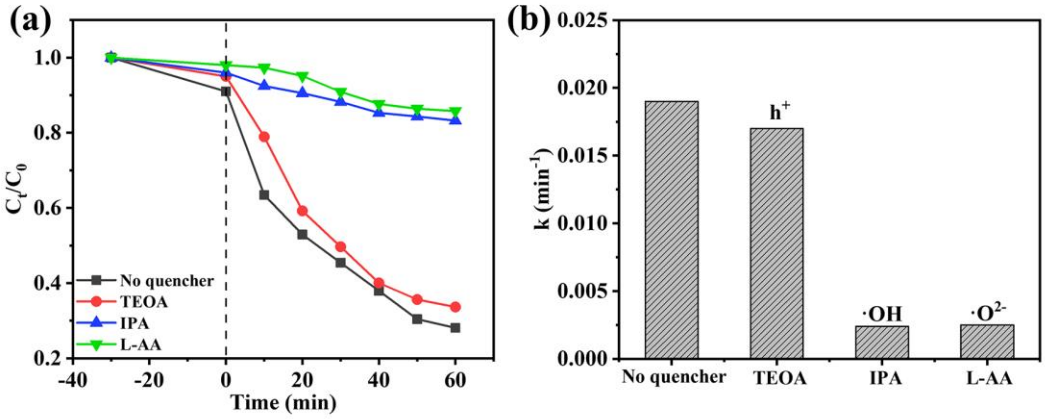

Figure S12. Photodegradation efficiencies in the presence of different quenchers.

References

1. Zhang, T. X.; Jiao, S.; Zhao, J. X.; Gao, G. R.; Yang, Y. Y.; Guo, C. L., Solar water evaporation using porous cellulose polyacrylamide hydrogel with carbon-based material containing copper oxide prepared from after-use adsorbent. *Desalination* **2022,** *527*, 115576.
2. Farid, M. U.; Kharraz, J. A.; Wang, P.; An, A. K., High-efficiency solar-driven water desalination using a thermally isolated plasmonic membrane. *J. Clean Prod.* **2020,** *271*, 122684.
3. Wang, Q. M.; Jia, F. F.; Huang, A. H.; Qin, Y.; Song, S. X.; Li, Y. M.; Arroyo, M. A. C., MoS_2@sponge with double layer structure for high-efficiency solar desalination. *Desalination* **2020,** *481*, 114359.
4. Han, J.; Xing, W. Q.; Yan, J.; Wen, J.; Liu, Y. T.; Wang, Y. Q.; Wu, Z. F.; Tang, L. C.; Gao, J. F., Stretchable and Superhydrophilic Polyaniline/Halloysite Decorated Nanofiber Composite Evaporator for High Efficiency Seawater Desalination. *Advanced Fiber Materials* **2022,** *4* (5), 1233-1245.
5. Wang, S.; Niu, Y.; Yan, L. J.; Chan, W. J.; Zhu, Z. Q.; Sun, H. X.; Li, J. Y.; Liang, W. D.; Li, A., Polyimide-based superhydrophilic porous membrane with enhanced thermal insulation for efficient interfacial solar evaporation. *Composites Science and Technology* **2022,** *228*, 109683.
6. Wu, C. M.; Cheng, C. T.; Tessema, A. A.; Motora, K. G.; Rani, G. M., Staple carbon fabric/polyurethane Janus membranes for photothermal conversion and interfacial steam generation. *J. Polym. Res.* **2023,** *30* (6).
7. Xi, Y. B.; Guo, W. Q.; Wang, X. J.; Lin, X. L.; Lyu, G., Photothermal Properties and Solar Water Evaporation Performance of Lignin-Based Polyurethane Foam Composites. *Langmuir* **2024,** *40* (13), 7205-7214.
8. Zhao, Y. N.; Liu, Z. X.; Yu, L.; Zhang, J. R.; Wu, F.; Lv, T. T.; Zhao, C.; Xing, G. J., Self-floating and long-term stable $Ti_3C_2T_x$/polyurethane composite membranes with highly efficient photothermal conversion performances for multiple applications. *Desalination* **2024,** *583*, 117720.
